# Characteristics and Range of Reviews About Technologies for Aging in Place: Scoping Review of Reviews

**DOI:** 10.2196/50286

**Published:** 2024-01-22

**Authors:** Jenny M Bergschöld, Mari Gunnes, Arne H Eide, Eva Lassemo

**Affiliations:** 1 Department of Health SINTEF Digital Trondheim Norway; 2 Department of Health SINTEF Digital Oslo Norway

**Keywords:** aging in place, technology, gerontechnology, assistive technology, gerontology, geriatric, geriatrics, older adult, older adults, aging, aging, scoping, review methods, review methodology, older people, evidence map, evidence mapping

## Abstract

**Background:**

It is a contemporary and global challenge that the increasing number of older people requiring care will surpass the available caregivers. Solutions are needed to help older people maintain their health, prevent disability, and delay or avoid dependency on others. Technology can enable older people to age in place while maintaining their dignity and quality of life. Literature reviews on this topic have become important tools for researchers, practitioners, policy makers, and decision makers who need to navigate and access the extensive available evidence. Due to the large number and diversity of existing reviews, there is a need for a review of reviews that provides an overview of the range and characteristics of the evidence on technology for aging in place.

**Objective:**

This study aimed to explore the characteristics and the range of evidence on technologies for aging in place by conducting a scoping review of reviews and presenting an evidence map that researchers, policy makers, and practitioners may use to identify gaps and reviews of interest.

**Methods:**

The review was conducted in accordance with the PRISMA-ScR (Preferred Reporting Items for Systematic Reviews and Meta-Analyses extension for Scoping Reviews). Literature searches were conducted in Web of Science, PubMed, and Scopus using a search string that consisted of the terms “older people” and “technology for ageing in place,” with alternate terms using Boolean operators and truncation, adapted to the rules for each database.

**Results:**

A total of 5447 studies were screened, with 344 studies included after full-text screening. The number of reviews on this topic has increased dramatically over time, and the literature is scattered across a variety of journals. Vocabularies and approaches used to describe technology, populations, and problems are highly heterogeneous. We have identified 3 principal ways that reviews have dealt with populations, 5 strategies that the reviews draw on to conceptualize technology, and 4 principal types of problems that they have dealt with. These may be understood as methods that can inform future reviews on this topic. The relationships among populations, technologies, and problems studied in the reviews are presented in an evidence map that includes pertinent gaps.

**Conclusions:**

Redundancies and unexploited synergies between bodies of evidence on technology for aging in place are highly likely. These results can be used to decrease this risk if they are used to inform the design of future reviews on this topic. There is a need for an examination of the current state of the art in knowledge on technology for aging in place in low- and middle-income countries, especially in Africa.

## Introduction

### Background

The World Health Organization (WHO) estimates that the global population aged 60 years and older will increase from 12% to 22% between 2015 and 2050, with the most dramatic increase in low- and middle-income countries (LMICs) [1]. This will change the age composition in populations globally. Demographic aging refers to shifts in the age composition of populations where the proportion of the population that consists of older people grows significantly. The fact that people are now living longer than ever and that they are expected to continue doing so is the result of positive developments in public health and survival [2]. Yet, demographic aging is also one of the key challenges of our time [3].

This concern is caused by how demographic aging will impact nation states. As people grow older, they tend to become increasingly reliant on both formal and informal care. For instance, older people are more likely to have functional limitations, need assistance with everyday tasks, and need medical care [4-6]. Moreover, older individuals have lower incomes, which compound the challenges of their increasing need for care [7]. As a result, nation states have a variety of systems in place to care for older people, including systems of shouldering the cost of that care.

In countries where welfare and care systems are heavily subsidized, demographic aging is predicted to lead to heavy financial strain and a decreased quality of life for older people, unless solutions that cater to the need to maintain good health and affordable health care into a longer set of retirement years are developed [8]. Still, the adverse consequences of demographic aging will be even greater in LMICs. In LMICs, welfare systems often function poorly or are nonexistent, meaning that the burden of caring for older people falls on families or on the older individuals to care for themselves. This has caused concerns that LMICs will “grow old before growing rich” [7].

To neutralize the overwhelming demand for health care, solutions are needed to enable older people maintain their health for longer and postpone or avoid disability and dependency [1,9,10]. Against this background, interest in technology that enables older people to age in place while maintaining their dignity and quality of life has grown rapidly over the past 2 decades [11].

Aging in place is a concept that refers to the shared responsibility of individuals and public authorities to enable older people to continue to live safely, relatively independently, and comfortably in the community either in their current home or in appropriate housing, regardless of age, income, or level of competence [1,12-14]. The idea is that policies and public services should address the challenges posed by demographic aging by finding alternatives to traditional forms of older adult care and creating solutions that are less resource-intensive. In welfare states where health and care services are heavily subsidized, this shift toward less resource-intensive solutions generally refers to options that maintain a high quality of life for older people while simultaneously preventing or delaying the need to relocate to a nursing home or becoming dependent on care [15], as well as to solutions that minimize the use of resources in nursing homes and other forms of formal care, without compromising their quality. Meanwhile, in LMICs, the main challenge is that welfare systems are weak and even nonexistent. Assistive technology and related services are marginal and not available for the majority, particularly for the poor rural populations. Ensuring assistive technology for all, including the growing number of older adults, requires resources and build-up of competence through a sustainable systems approach [16]. In this context, innovations are needed in service delivery, and community-based models as well as adaptation of existing assistive technology and development of new and contextually relevant assistive technology are needed to ensure that older people live well and as autonomously as possible [17].

Technologies that enable aging in place encompass a wide variety of technologies designed to monitor or support the health and activities of older people or strengthen their contact with others [11,15]. In some cases, older people are the intended users, but technology can also be used to establish links between older adults and their circles of care. Technologies for aging in place include both high- and low-tech solutions, including but not limited to mobility devices, information and communication technologies, assistive technologies, sensor technology, telemedicine, health monitoring, games, wearables and medication reminders, and the internet of things [15,18-27].

### Rationale

Alongside the interest in technology that can enable older people to age in place, the number of publications on this topic has increased dramatically. In this context, literature reviews can be important tools for researchers as well as practitioners, policy makers, and decision makers who need to navigate current debates and access syntheses of the available evidence. Yet, to date, there is no review of the available published reviews that provide an overview of the range and characteristics of the evidence on technology for aging in place.

While reviews of reviews on technologies for aging in place do exist, they typically limit the scope to health conditions, diseases, technologies, or caring practices, for instance, by focusing on the self-efficacy of older people using technology to self-manage chronic obstructive pulmonary disease, hypertension, heart failure, or dementia at home [28]; on the effects of digital technologies on older people’s access to health and social care [29]; on the promotion of physical activity in older people using mobile health (mHealth) and eHealth technologies [30]; or on how mHealth technology may support aging in place [31] and procedures of user-centered usability assessment for digital solutions [32].

### Objective

The objective of this review of reviews is to explore the characteristics and the range of evidence on technologies for aging in place by conducting a scoping review of reviews in accordance with the PRISMA-ScR (Preferred Reporting Items for Systematic Reviews and Meta-Analyses extension for Scoping Reviews) [33]. The PRISMA-ScR checklist is available in Multimedia Appendix 1.

By exploring the included reviews, we are particularly interested in what year and in which journals they are published, which review methods that characterize reviews in this field, and whether there are any reviews that are explicitly concerned with LMICs. By LMICs, we mean the countries identified by the Organization for Economic Co-operation and Development as having low-income or middle income economies, which may be updated from time to time by the Organization for Economic Co-operation and Development [34]. In exploring the range of evidence presented in reviews on technologies for aging in place, we are particularly interested in which types of populations, technologies, and problems they have been concerned with.

## Methods

### Eligibility Criteria

We included literature reviews in English about technology for older people or older adult care, including informal care, that we were able to access. To ensure the quality of our sources, we limited our scope to peer-reviewed literature reviews that have been published in academic journals. For the same reason, we only included reviews where the methods were clearly described. We did not apply any limits to the year of publication.

### Information Sources

Our method of selecting databases included making a list of the most relevant journals in the field that the authors were aware of (Multimedia Appendix 2). The complete list was sent to a panel of experts consisting of members from the WHO and the International Society of Gerontechnology, who were asked to add any potentially relevant journals missing from the list. After the list was considered complete, the authors identified the databases where these journals were indexed. The final selection of databases was Web of Science (Table 1), PubMed (Table 2), and Scopus (Table 3). The searches in Web of Science and Scopus were conducted on September 13, 2022, and the search in PubMed was conducted on September 14, 2022.

**Table 1:** Web of Science—core collection (n=1741).

	Search	Results
1	(((((((((((TS=(“old* per*”)) OR TS=(“old* peo*”) OR TS=(“old* age*”)) OR TS=(“old* adu*”) OR TS=(“old* use*”)) OR TS=(geriatric)) OR TS=(“aged per*”)) OR TS=(“aged peo*”) OR TS=(“aged use*”) OR TS=(ag$ing)) OR TS=(elder*)) OR TS=(senior)) OR TS=(retire*)) OR TS=(pension*)) OR TS=(“later life”))	4,027,248
2	(TS=(ai) OR TS=(“ag$ in place”) OR TS=(gerontechnology) OR TS=(“assisted living”) OR TS=(“assist* tech”) OR TS=(assist* device*) OR TS=(“tele*”) OR TS=(“welfare tech*”) OR TS=(“digital* health”) OR TS=(“digital* care”) OR TS=(“smart hom*”) OR TS=(“smart hea*”) OR TS=(“mobile health”) OR TS=(mhealth) OR TS=(ehealth) OR TS=(robot*))	1,067,363
3	#6 AND #5 and Review Article (Document Types) and English (Languages)	1741

**Table 2:** PubMed (n=2402).

	Search	Results
1	(“old per*”[Title/Abstract] OR “old peo*”[Title/Abstract] OR “old adu*”[Title/Abstract] OR “old use*”[Title/Abstract] OR “geriatric”[Title/Abstract] OR “aged pe*”[Title/Abstract] OR “aging”[Title/Abstract] OR “ageing”[Title/Abstract] OR “elder*”[Title/Abstract] OR “senior”[Title/Abstract] OR “retire*”[Title/Abstract] OR “pension*”[Title/Abstract] OR “later life”[Title/Abstract]) AND (english[Filter])	584,813
2	(“ai”[Title/Abstract] OR “aging in place”[Title/Abstract] OR “ageing in place”[Title/Abstract] OR “gerontechnology”[Title/Abstract] OR “assisted living”[Title/Abstract] OR “assistive living”[Title/Abstract] OR “assist* tech*”[Title/Abstract] OR “assist* device*”[Title/Abstract] OR “tele*”[Title/Abstract] OR “welfare tech*”[Title/Abstract] OR “digital health”[Title/Abstract] OR “digital care”[Title/Abstract] OR “smart hom*”[Title/Abstract] OR “smart hea*”[Title/Abstract] OR “mobile health”[Title/Abstract] OR “mhealth”[Title/Abstract] OR “ehealth”[Title/Abstract] OR “robot*”[Title/Abstract]) AND (english[Filter])	319,264
3	(“independent living”[MeSH Terms] OR “self help devices”[MeSH Terms] OR “artificial intelligence”[MeSH Terms] OR “telemedicine”[MeSH Terms]) AND ((review[Filter]) AND (english[Filter]))	16,843
4	(“aged”[MeSH Terms] OR “aging”[MeSH Terms]) AND ((review[Filter]) AND (english[Filter]))	11,092
5	#1 AND (#2 OR #3 OR #4)	2402

**Table 3:** SCOPUS (n=3131).

#	Search	Results
1	TITLE-ABS-KEY ( ( “old* per*” ) OR ( “old* peo*” ) OR ( “old* age*” ) OR ( “old* adu*” ) OR ( “old* use*” ) OR ( geriatric ) OR ( “aged per*” ) OR ( “aged peo*” ) OR ( “aged use*” ) OR ( ag*ing ) OR ( elder* ) OR ( senior ) OR ( retire* ) OR ( pension* ) OR ( “later life” ) )	2,282,529
2	TITLE-ABS-KEY ( ( ai OR “ag* in place” OR gerontechnology OR “assisted living” OR ( “assist* tech” ) OR ( “assist* device*” ) OR tele* OR ( “welfare tech*” ) OR ( “digital* health” ) OR ( “digital* care” ) OR ( “smart hom*” ) OR ( “smart hea*” ) OR ( “mobile health” ) OR mhealth OR ehealth OR robot* ) )	2,047,409
3	(LIMIT-TO (DOCTYPE, “re” ) ) AND ( LIMIT-TO ( LANGUAGE , “English” ) )	3131

### Search

A search consisting of the terms “older people” and “technology for ageing in place” with alternate terms was conducted using Boolean operators and truncation. The search was adapted to the rules for each database.

### Selection of Sources of Evidence

**Figure 1:**
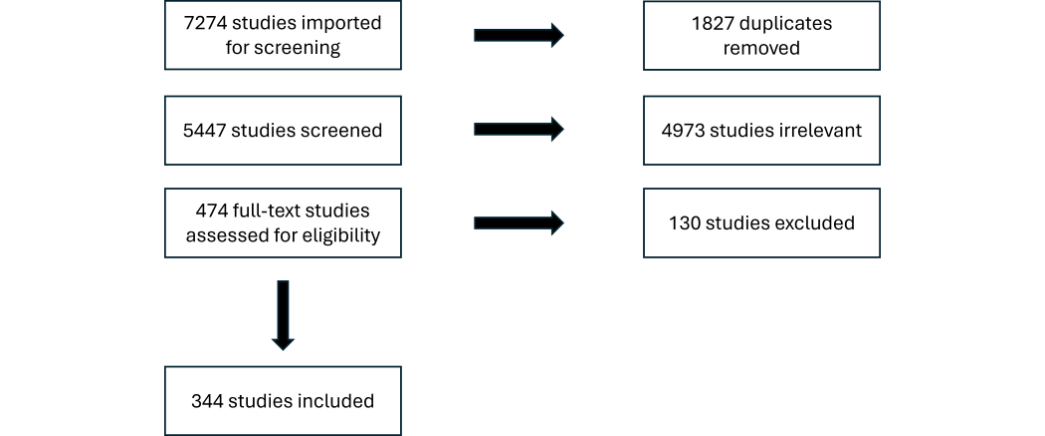
Screening process flowchart.

The search resulted in a total of 7274 identified studies, that is, 3131 from Scopus, 2402 from PubMed, and 1741 from Web of Science. We used Covidence (Veritas Health Innovation) to organize the review process. After 1827 duplicates were identified and removed, 5447 studies were screened using the eligibility criteria (see Textbox 1). The original list of eligibility criteria contained items 1-7. However, after we identified a retracted paper, we decided to add exclusion criterion 8 “retracted paper.” The title and abstract screening resulted in the exclusion of 4973 studies. The full-text screening resulted in the further exclusion of 130 studies, and the remaining 344 studies were included in the data charting process. Figure 1 illustrates this process.

**Eligibility criteria.**

**Inclusion criteria**

- Literature reviews
- Journal paper
- Peer-reviewed research
- Able to source full text
- Methodologically sound
- About technology for aging in place
- English

**Exclusion criteria, with a short label for Covidence**

- Not a literature review—Papers that do not review the literature
- Not a journal paper—Anything that is not a paper meaning: book chapters, conference proceedings, protocols, reports, preprints, etc
- Not research—editorials, opinion pieces, press, etc
- Unable to source—currently unable to access full text currently
- Method not described—Reviews that do not clearly describe their methods
- Thematically irrelevant—Not about technology for aging in place
- Not in English
- Retracted paper

### Data Charting Process and Data Items

The data were extracted using the data extraction template feature in Covidence. The extraction of data was organized in line with our objectives and research questions. Tables 4 and 5 provide an overview of the relationship between the research questions and the extracted data.

**Table 4:** Research questions and charted data that relate to the characteristics of reviews on technologies for aging in place.

Research question	Extracted data
During what years were the reviews published?	Year of publication
In which journals have the reviews been published?	The name of the journal where they are published
Which review methods characterize the reviews?	The named review methods they refer to
Is there an explicit concern with LMICs?^a^	If they refer explicitly to LMICs (yes or no)

^a^LMIC: low- and middle-income countries.

**Table 5:** Research questions and charted data that relate to the range of evidence in reviews on technologies for aging in place.

Research question	Extracted data
Which populations are they concerned with?	The population specified in the review
Which types of technology are they concerned with?	The technology specified in the review
What type of problems are they concerned with?	The issues of interest specified in the review
What is the relationship between the populations, problems, and technologies the reviews have dealt with	The type of technology and the issues of interest specified in the review

All authors participated in the choice of databases and establishing the search terms and eligibility criteria. EL constructed the search string and conducted the final search. All authors participated in the screening process. The full-text papers were extracted by the authors JMB, MG, and AHE. All authors participated in the data synthesis and presentation of the findings.

## Results

### Characteristics of Evidence on Technologies for Aging in Place

Multimedia Appendix 3 shows an overview of the data and sources that correspond to this section. The number of reviews of evidence on technology for aging in place has increased dramatically over the past few years (Table 6). The earliest review included in our search was published in 2001 [35]. A total of 20 reviews were published between 2001 and 2010. By comparison, 142 reviews were published between 2015 and 2020. Note that the table only includes reviews published before September 13, 2022, when our search was conducted.

From 2020 to 2021, there was a near doubling in number of reviews. Since our search was conducted at the beginning of September 2022, the figure does not show the full extent of published reviews in 2022. However, it is likely that the trend will continue upwards. The included reviews were published in 183 unique journals. Of those, most journals have only published 1 or 2 reviews since 2001. Only 12 journals have published more than 5 reviews in total since 2001 (Table 7).

**Table 6:** Number of reviews by year of publication (n=344).

Year of publication	Reviews, n
2001	1
2002	0
2003	0
2004	1
2005	1
2006	0
2007	5
2008	4
2009	6
2010	2
2011	4
2012	8
2013	12
2014	14
2015	6
2016	16
2017	23
2018	23
2019	33
2020	47
2021	88
2022	50

**Table 7:** Overview of 12 journals that have published 5 or more reviews on technologies for aging in place since 2001.

Journals	Reviews, n
*Clinical Interventions in Aging*	5
*Healthcare*	5
*Assistive Technology*	6
*Journal of Telemedicine and Telecare*	7
*Maturitas*	8
*JMIR Aging*	10
*International Journal of Environment Research and Public Health*	11
*Sensors*	11
*The Gerontologist*	11
*Disability and Rehabilitation: Assistive Technology*	13
*International Journal of Medical Informatics*	16
*Journal of Medical Internet Research*	17

The reviews refer to 15 unique types of review methods. Of these, the most common were systematic reviews (n=144) and scoping reviews (n=60). The third most common review method was to provide a detailed account of the procedures but refrain from referring to a specific type of review method (n=98). While there were only 13 integrative reviews and 6 narrative reviews, the fact that most other review methods only occurred once or twice made the narrative reviews common by comparison (Table 8).

**Table 8:** Overview of the data analysis methods used in the included reviews.

Data analysis method	Reviews, n
Systematic review	144
Scoping review	60
Integrative review	13
Narrative	6
Mini-review	5
Review of reviews	5
Rapid review	2
Umbrella review	1
Targeted review	1
Meta-interpretive review	1
Focused literature review	1
Descriptive review	1
Clinical review	1
Critical interpretive synthesis	1
Conceptual review	1
Comprehensive review	1
Comparative literature review	1
Reflective review	1
Unspecified	98

Only 1 review referred explicitly to LMICs [36]. This review aimed to identify policy gaps in the delivery and availability of assistive health technology and medical devices for aging populations, particularly in LMICs, and found that practical, life-enhancing support for older people through assistive health technology, medical technology, and related health and social services is a neglected issue.

### Range of Evidence on Technologies for Aging in Place

#### Populations

Multimedia Appendix 4 shows an overview of data and sources that correspond to this section. Some reviews dealt with more than one type of population.

The included reviews dealt with populations in three ways by (1) describing the population in terms of older people or different types of caring roles (n=253), (2) describing the population in terms of a particular health condition or diagnosis (n=73), or (3) not specifying the population (n=43).

Of the included reviews, 253 described the population in terms of people and the roles they play in the context of aging. Of those reviews, an overwhelming majority only included studies on older people (n=220). Of the included reviews, 12 reviews dealt with formal and informal caregivers or combinations of these 3 different populations (Table 9).

**Table 9:** Overview of the populations in 253 reviews that described the population in terms of people and the roles they play in the context of aging.

Populations	Reviews, n
Older people	220
Formal caregivers	1
Formal caregivers and informal caregivers	2
Informal caregivers	5
Older people and formal caregivers	5
Older people, formal caregivers, and informal caregivers	4
Older people and informal caregivers	16

In total, 73 reviews described the population in terms of older people as well as individuals from other age groups, with a particular diagnosis or health problem. These reviews included studies about people of different age groups with different cognitive impairments exclusively (n=41) or in combination with other health problems (n=2). Notably then, these reviews included evidence based on studies of younger people as well as older people. Table 10 provides an overview of the diagnoses and health problems that these reviews used to conceptualize the populations.

In total, 43 reviews did not specify the population at all. Instead, they referred to the context of aging in place. These reviews were typically concerned with the technical functionality of devices rather than the interplay between what the technology offers and the intended users and their problems.

**Table 10:** Overview of the diagnoses and health problems used to conceptualize the population in reviews about older people and others with a particular diagnosis or health problem (in total n=73 reviews).

Diagnoses and health problems	Reviews, n
Cancer	1
Cardiovascular diseases	1
Cardiovascular diseases, diabetes, and asthma	1
Chronic conditions	7
Cognitive impairments	41
Cognitive impairments, cardiovascular diseases, and chronic obstructive pulmonary disease	1
Cognitive impairments, neurological disorders, falls, and cardiovascular disease	1
Complex needs	1
Decline in hand grip and dexterity	1
Diabetes	1
Falls	4
Falls and frailty	1
Frailty	6
Frailty and decreased hearing	1
Hip injuries	1
Loneliness and social isolation	2
Mental health	2

#### Types of Technology

Multimedia Appendix 5 shows an overview of the data and sources that correspond to these results. Some reviews dealt with more than one type of technology.

We identified 69 different types of technology that reviews have been concerned with and 5 substantive strategies that the reviews have used to conceptualize the technology with which they are concerned. Two of the 345 reviews used other strategies for conceptualizing technology. One was about co-designed technologies [37]. The other was about what they termed as consumer technology as well as smart environments [38].

The first and most common strategy (n=140 reviews) is to refer to technology by using descriptive technical terms such as “sensors” [39-48], “artificial intelligence” [49-52], “GPS” [53-55], or “games” [56-60]. The reviews that used this strategy covered 31 different types of technology exclusively or in combination with each other. Most of these reviews were focused on robots or robopets (n=47), information and communication technology (n=23), smart environments (n=17), or sensors (n=10).

The second strategy (n=65) is to conceptualize technology by the purpose of the technology in relation to a disease or type of health challenge that the technology addresses or is believed to be able to address, for instance, by using terms such as “technology for dementia” [61-70], “technologies for social connectedness” [71-75], “technology for frailty” [76-78], “technology for safety” [79], or “technology for falls” [60,80-84]. Most of the reviews that relied on this strategy dealt with assistive technologies (n=28).

The third strategy (n=30 reviews) is to refer to technologies in terms of their intended purpose in caring services or practices that they are part of. Such terms include “teleopthamology” [85], “monitoring technologies” [86,87], “telerehabilitation” [88-93], “technology for home health care” [94,95], or “technology for pain management” [96]. Most reviews that relied on this strategy were concerned with telerehabilitation (n=7) or technology for health information (n=6).

The fourth strategy (n=29 reviews) is to describe the type of technology by using umbrella terms that broadly refer to the use of technology to enable older people to age in place, for instance, by defining the technology of interest in terms of “technology for ageing in place” [11,97-99], “gerontechnology” [100-102], “welfare technology” [103-105], “technology for healthy ageing” [106,107], or “technology for older people” [22,32,108-112].

The fifth strategy (n=75 reviews) is to describe the type of technology the review is concerned with by way of concepts that refer to the use of technology as part of a broad range of caring services, strategies, and practices, such as, for instance, telecare [113-119], telemedicine [120-125], e-interventions [126], or eHealth [127-143]. Most reviews that used this type of concept to describe the technology they are concerned with dealt with mHealth (n=18), eHealth (n=17), or telehealth (n=18).

#### What Types of Problems Have the Reviews Dealt With?

Multimedia Appendix 6 shows an overview of the corresponding data and sources. Some reviews dealt with more than one type of problem. We identified 49 unique problem topics and 4 principal types of problems.

The first type of problem is related to different types of care services or caring practices (n=60 reviews). Most of these reviews dealt with problems related to the context of home care (n=30), caring practices in nursing homes or other long-term care institutions (n=11), or rehabilitation (n=7). By contrast, other topics occurred only once or twice, that is, problems related to caregiver burden [144,145], dementia care [146], emergency care services [147], informal care [148], and health information services [149,150].

The second type of problem is issues related to the management of health-related issues or diseases in the context of aging in place (n=128 reviews). Of those, most dealt with problems related to cognitive impairments either exclusively (n=61) or in combination with one or several other health problems (n=10), that is, cognitive impairment and mental health [151-155], or cognitive impairment, stroke, cardiovascular disease, and falls [156]. Other problems that were featured relatively frequently included falls and balance–related issues (n=19), frailty (n=8), chronic conditions (n=8), and depression (n=5). Meanwhile, other problems related to the management of other health-related issues and diseases featured only once or twice, despite being common health challenges for older people (for instance, Parkinson’s disease [157,158], malnutrition [159], dental health [160], eye diseases [85], and pain [96,161]).

The third type of problem relates to the experience of aging in place (n=82 reviews). The most common topics in this category were loneliness, including social isolation or connectedness (n=21 reviews), older peoples’ self-care or self-management (n=19 reviews), and active aging (n=16 reviews). Other topics in this category include healthy aging [106,111,137,162-165], information needs [166], quality of life [97,167-173], quality of life and older people’s self-care and self-management [174,175], and activities of daily living exclusively [176-179] or in combination with other topics such as loneliness [180,181], or quality of life [182].

The fourth type of problem relates to the research and development of technology. This was the most common type of problem (n=285 reviews). The overwhelming majority of reviews that dealt with this type of problem were concerned with barriers and drivers of use and acceptability (n=114), the effect or implications of technology (n=86), or the combination of these 2 topics (n=21). Other common topics included uptake or scalability (n=15), user involvement (n=11), ethical considerations (n=14), feasibility (n=10), and cost-effectiveness or use (n=7).

Notably, problems related to home care (n=30), loneliness (n=21), cognitive impairments (n=71), barriers and drivers of use and acceptability (n=114 reviews), and the effect or implications of technology (n=86) have been heavily emphasized. Meanwhile, others such as cost-effectiveness or use of technologies (n=7), health information needs (n=1), malnutrition (n=1), dental health (n=1), eye diseases (n=1), and pain management seem underprioritized by comparison.

#### What Are the Relationships Between the Problems, Technologies, and Populations That the Reviews Have Dealt With

Multimedia Appendix 7 shows an evidence map that provides an overview of the relationships between problems, technologies, and populations that the reviews have been concerned with. Some reviews deal with more than one population, technology, and type of problem. Multimedia Appendix 8 shows an overview of the corresponding data and sources.

As illustrated in the evidence map (Multimedia Appendix 7), many reviews draw on an evidence base that is not specific to older people or their caregivers.

This is particularly notable in the reviews on the following topics: barriers and drivers of use and acceptability, cognitive impairment, and the effect or implications of technology. The same observation applies to the following types of technology such as assistive technologies, robots, technology for dementia, technology for falls, technology for frailty, telehealth, and technology for Alzheimer disease.

### Summary of Evidence

In exploring the range and characteristics of reviews on technology for aging in place, we found that the number of reviews, as well as the pace at which they are published, has increased dramatically over time. While some journals such as *JMIR Aging*, *Disability and Rehabilitation: Assistive Technology*, the *Journal of Medical Internet Research*, and *The Journal of Medical Informatics* have published more reviews on this topic than others, the literature is scattered over 183 unique journals. Most reviews on this topic are systematic reviews (n=144).

In exploring the range of reviews on technology for aging in place, we identified 3 principal ways that reviews have dealt with populations. Specifically, the 3 ways are describing the population in terms of older people or different types of caring roles (n=253), in terms of people affected with a particular health condition or diagnosis (n=73), or not specifying the population (n=43). These may be considered as methods of conceptualizing populations. We identified 88 unique types of technology that the reviews have dealt with. We also found that there are strong tendencies for reviews to synthesize the evidence on broad and unspecific categories of technology such as “ICT” or “robots” rather than to concentrate on a particular device (a notable exception is a review on personal alarms [183]). Moreover, we identified 5 strategies that the reviews draw on to conceptualize technology. Those strategies are to (1) refer to technology by using descriptive technical terms; (2) conceptualize technology by way of the purpose of the technology about a disease or health issue; (3) refer to technologies in terms of their purpose in caring services or practices; (4) use umbrella terms that broadly refer to the use of technology to enable older people to age in place; and (5) use concepts that refer to the technology as part of caring services, strategies, and practices. We also identified 4 principal types of problems and 49 unique subtypes of problems that the reviews have dealt with. The four principal types are problems related to (1) different types of care services or caring practices, (2) the management of health problems or diseases, (3) the experience of aging in place, and (4) the research and development of technology. The evidence map (Multimedia Appendix 6) demonstrates the relationships between the populations, technologies, and problems studied in the reviews and illustrates the gaps. Notably, many of the reviews on the most studied technologies and problems draw on studies that are not specific to older people or the context of aging in place, either by not specifying the population at all or by including studies on patients of all ages, meaning that topics studied only by such reviews should also be considered gaps.

## Discussion

**Summary of Evidence**

Together, these results speak to the need for regularly updated overviews of ongoing debates in the field. However, they are also illustrative of the challenges that such overviews must overcome. For instance, the lack of conceptual hegemony means that any attempt to describe the technologies that the reviews have been concerned with in purely technical terms fails to grasp the diverse ways that technology is understood in this field. A more fruitful approach is to categorize them according to the different ways that they understand and deal with technology. Used as methodological tools, the strategies of defining populations, conceptualizing technology, the typology of problems, and the overview of the relationships presented here can inform the design of future reviews and enable researchers to purposefully identify gaps and publications that are likely to be of relevance to each other despite conceptual differences that may obscure their similarities.

It is notable that only 1 review was explicitly concerned with LMICs, considering that the greatest growth in older people globally will be in LMICs [1], particularly in Africa where the population of 60 years and older is expected to increase by more than 100% by 2050 [184]. Similarly, it is notable that in the included reviews, relatively little attention has been paid to formal and informal caregivers. Both formal and informal caregivers play important roles in the context of technology for aging in place. Both formal and informal caregivers frequently speak and act on behalf of older people, especially older people with cognitive impairments when technology developers seek to identify user needs or evaluate the usefulness of the technology [185-188]. In doing so, they act as gatekeepers who shape what types of technology are developed and offered to older people, and equally important, which are not [109]. Both formal and informal caregivers are often the intended users of technology that is meant to enable older people to age in place. Thus, the politics of their lives and working conditions as well as the quality and type of care they are able to provide to older people are shaped by what the technology affords and prohibits [189-191]. Yet, the purpose of the technology is aimed at the needs of the older person or efficiency-related goals in care organizations rather than the improvement of the care workers’ working environment or care burden. Additionally, like all users, both formal and informal caregivers are not just impacted by technologies that enter their lives but they also shape the technology in turn [188,192-199], meaning that the implications that the technology will have in practice are never given beforehand and must always be studied in the context of use [185,187,200,201]. Finally, both informal and formal caregivers must frequently improvise and adapt the technology to render it functional [192,202-205]. Thus, both formal and informal caregivers play important roles in shaping the practices, politics, and services that the technology affords or delimits in the lives of older people who age in place. These roles have been thoroughly described in the literature. Yet, they seem overlooked in reviews on technology for aging in place.

It is problematic that so many reviews concerned with problems related to technologies for aging in place draw on an evidence base that is not specific to older people. Older people frequently have other needs than younger people even when they share a diagnosis because the aging body presents specific challenges, which increase the risk of illnesses, falls, disability, and death [206]. It is therefore unlikely that reviews that do not focus explicitly on older people are able to grasp and address the specificity of the challenges that older people face as part of aging in place. This primarily concerns reviews on the topics of barriers and drivers of use and acceptability, cognitive impairment, and the effect or implications of technology. It also concerns reviews about assistive technologies, robots, technology for dementia, technology for falls, technology for frailty, telehealth, and technology for Alzheimer disease. While these topics and technologies have frequently been addressed, the value that reviews that do not specify their population or that base their arguments on studies of people of all ages (see Multimedia Appendix 6) is limited, and there is a need for more targeted and age-specific syntheses reviews to better address the unique requirements of older individuals and their caregivers. The strong tendency for reviews in this field to concentrate on broad and unspecific categories of technology, such as “ICT” or “robots” means that there is no straightforward way for practitioners to use these reviews as support in decision-making processes regarding the potential usefulness and challenges related to specific devices*.*

### Limitations

Despite the many methodological strengths of the design of a scoping review of reviews, there are some limitations to be considered. These include the potential for bias in the review process, the difficulty ensuring the quality and reliability of the included reviews, and the potential for the review to be influenced by the perspectives and priorities of the researchers conducting the review. Considering the broad eligibility criteria chosen for this review, the results may be considered representative of the characteristics and range of evidence on technologies for aging in place. However, the inclusion of more databases could have expanded the data set even further, and potentially relevant literature that does not use the term aging in place explicitly may have been missed. Moreover, this review has not sought to explore or synthesize the results of the included reviews nor have we considered the quality of the included reviews.

### Conclusions

The number of published reviews on this topic in the past few years in combination with the rate at which they are published suggests that redundancies and a lack of fruitful synergies between them are likely. The breadth of variation concerning how reviews have dealt with populations, conceptualizations of types of technology, and problems demonstrates the conceptual differences that must be bridged to remedy this problem.

Together, these results underscore the necessity for improved coordination and collaboration among reviews while also recognizing the potential benefits of more standardized vocabularies.

The insights gained from the methods of dealing with populations, strategies for conceptualizing types of technology, and the types of problems identified in this study may be used methodologically to identify commonalities and connections that may otherwise be obscured by differing conceptual frameworks.

There is an urgent need for an examination of the current state of the art in knowledge regarding technology for aging in place in LMICs. Developing a deeper understanding of the conditions surrounding aging in LMICs, especially in Africa, and the implications those conditions have for the roles that technology may play and not play in the lives of older people and their circles of care should be an essential focus of the research agenda.

## Appendix

Multimedia Appendix 1 PRISMA-ScR Checklist.

Multimedia Appendix 2 Journals to guide the selection of databases.

Multimedia Appendix 3 Characteristics of evidence on technologies for ageing in place.

Multimedia Appendix 4 Populations.

Multimedia Appendix 5 Types of technology.

Multimedia Appendix 6 Types of problems.

Multimedia Appendix 7 Evidence map.

Multimedia Appendix 8 Relationships between problems technologies and populations.

## Abbreviations

LMIC: low- and middle-income country
mHealth: mobile health
PRISMA-ScR: Preferred Reporting Items for Systematic Reviews and Meta-Analyses extension for Scoping Review
WHO: World Health Organization
