# Supplementary material for: Characteristics and Range of Reviews About Technologies for Aging in Place: Scoping Review of Reviews
Source: JMIR Aging. 2024 Jan 22;7:e50286. doi: 10.2196/50286 (PMC10845034; doi:10.2196/50286)
Supplement: Multimedia Appendix 1 [file aging_v7i1e50286_app1.docx]

**Preferred Reporting Items for Systematic reviews and Meta-Analyses extension for Scoping Reviews (PRISMA-ScR) Checklist**

| **SECTION** | **ITEM** | **PRISMA-ScR CHECKLIST ITEM** | **REPORTED ON PAGE #** |
| --- | --- | --- | --- |
| **TITLE** | | | |
| Title | 1 | Characteristics and Range of Reviews About Technologies for Aging in Place: Scoping Review of Reviews. | 1 |
| **ABSTRACT** | | | |
| Structured summary | 2 | **Background:** It is a contemporary and global challenge that the increasing number of older people requiring care will surpass the available caregivers. Solutions are needed to help older people maintain their health, prevent disability, and delay or avoid dependency on others. Technology can enable older people to age in place while maintaining their dignity and quality of life. Literature reviews on this topic have become important tools for researchers, practitioners, policy makers, and decision makers who need to navigate and access the extensive available evidence. Due to the large number and diversity of existing reviews, there is a need for a review of reviews that provides an overview of the range and characteristics of the evidence on technology for aging in place.  **Objective:** This study aimed to explore the characteristics and the range of evidence on technologies for aging in place by conducting a scoping review of reviews and presenting an evidence map that researchers, policy makers, and practitioners may use to identify gaps and reviews of interest.  **Methods:** The review was conducted in accordance with the PRISMA-ScR (Preferred Reporting Items for Systematic Reviews and Meta-Analyses extension for Scoping Reviews). Literature searches were conducted in Web of Science, PubMed, and Scopus using a search string that consisted of the terms “older people” and “technology for ageing in place,” with alternate terms using Boolean operators and truncation, adapted to the rules for each database.  **Results:** A total of 5447 studies were screened, with 344 studies included after full-text screening. The number of reviews on this topic has increased dramatically over time, and the literature is scattered across a variety of journals. Vocabularies and approaches used to describe technology, populations, and problems are highly heterogeneous. We have identified 3 principal ways that reviews have dealt with populations, 5 strategies that the reviews draw on to conceptualize technology, and 4 principal types of problems that they have dealt with. These may be understood as methods that can inform future reviews on this topic. The relationships among populations, technologies, and problems studied in the reviews are presented in an evidence map that includes pertinent gaps.  **Conclusions:** Redundancies and unexploited synergies between bodies of evidence on technology for aging in place are highly likely. These results can be used to decrease this risk if they are used to inform the design of future reviews on this topic. There is a need for an examination of the current state of the art in knowledge on technology for aging in place in low- and middle-income countries, especially in Africa. | 1 |
| **INTRODUCTION** | | | |
| Rationale | 3 | Alongside the interest in technology that can enable older people to age in place, the number of publications on this topic has increased dramatically. In this context, literature reviews can be important tools for researchers as well as practitioners, policy makers, and decision makers who need to navigate current debates and access syntheses of the available evidence. Yet, to date, there is no review of the available published reviews that provide an overview of the range and characteristics of the evidence on technology for aging in place. | 2 |
| Objectives | 4 | The objective of this review of reviews is to explore the characteristics and the range of evidence on technologies for aging in place by conducting a scoping review of reviews | 3 |
| **METHODS** | | | |
| Protocol and registration | 5 | No. | - |
| Eligibility criteria | 6 | We included literature reviews in English about technology for older people or eldercare, including informal care, that we were able to access. To ensure the quality of our sources, we limited our scope to peer-reviewed literature reviews that have been published in academic journals. For the same reason, we only included reviews where the methods were clearly described. We did not apply any limits to the year of publication. | 3 |
| Information sources* | 7 | The final selection of databases was Scopus, Web of Science, and PubMed. Most recent search conducted Sept 14t 2023. | 4-5 |
| Search | 8 | See tables 1-3 | 4-5 |
| Selection of sources of evidence† | 9 | See Fig 1. | 5 |
| Data charting process‡ | 10 | See Tables 4-5 | 6 |
| Data items | 11 | See Tables 4-5. | 6 |
| Critical appraisal of individual sources of evidence§ | 12 | See Textbox 1 | 3 |
| Synthesis of results | 13 | See Tables 4-5 and Appendix 6 | 6 |
| **RESULTS** | | | |
| Selection of sources of evidence | 14 | See fig 1. | 5 |
| Characteristics of sources of evidence | 15 | See Appendices 2-5. | - |
| Critical appraisal within sources of evidence | 16 | If done, present data on critical appraisal of included sources of evidence (see item 12). | - |
| Results of individual sources of evidence | 17 | See Appendices 2-5 | - |
| Synthesis of results | 18 | See appendices 2-5 | - |
| **DISCUSSION** | | | |
| Summary of evidence | 19 | Summarize the main results (including an overview of concepts, themes, and types of evidence available), link to the review questions and objectives, and consider the relevance to key groups. | 12-13 |
| Limitations | 20 | Discuss the limitations of the scoping review process. | 13 |
| Conclusions | 21 | Provide a general interpretation of the results with respect to the review questions and objectives, as well as potential implications and/or next steps. | 13-14 |
| **FUNDING** | | | |
| Funding | 22 | Describe sources of funding for the included sources of evidence, as well as sources of funding for the scoping review. Describe the role of the funders of the scoping review. | 14 |

JBI = Joanna Briggs Institute; PRISMA-ScR = Preferred Reporting Items for Systematic reviews and Meta-Analyses extension for Scoping Reviews.

* Where *sources of evidence* (see second footnote) are compiled from, such as bibliographic databases, social media platforms, and Web sites.

† A more inclusive/heterogeneous term used to account for the different types of evidence or data sources (e.g., quantitative and/or qualitative research, expert opinion, and policy documents) that may be eligible in a scoping review as opposed to only studies. This is not to be confused with *information sources* (see first footnote).

‡ The frameworks by Arksey and O’Malley (6) and Levac and colleagues (7) and the JBI guidance (4, 5) refer to the process of data extraction in a scoping review as data charting*.*

§ The process of systematically examining research evidence to assess its validity, results, and relevance before using it to inform a decision. This term is used for items 12 and 19 instead of "risk of bias" (which is more applicable to systematic reviews of interventions) to include and acknowledge the various sources of evidence that may be used in a scoping review (e.g., quantitative and/or qualitative research, expert opinion, and policy document).

*From:* Tricco AC, Lillie E, Zarin W, O'Brien KK, Colquhoun H, Levac D, et al. PRISMA Extension for Scoping Reviews (PRISMAScR): Checklist and Explanation. Ann Intern Med. 2018;169:467–473. [doi: 10.7326/M18-0850](http://annals.org/aim/fullarticle/2700389/prisma-extension-scoping-reviews-prisma-scr-checklist-explanation).
