# Supplementary material for: Characteristics and Range of Reviews About Technologies for Aging in Place: Scoping Review of Reviews
Source: JMIR Aging. 2024 Jan 22;7:e50286. doi: 10.2196/50286 (PMC10845034; doi:10.2196/50286)
Supplement: Multimedia Appendix 2 [file aging_v7i1e50286_app2.pdf]

Journal	Index info	Database
Journal of Gerontechnology	(indexed by scopus but also others, index info here: https://journal.gerontechnology.org/about.aspx)	Scopus
Sociology of health and illness	list of index info here: https://onlinelibrary.wiley.com/page/journal/14679566/homepage/productinformation.html	PubMed
Technological forecasting and social change	(Elsevier, indexed by science direct)	Scopus
The gerontologist	(indexed by: Abstracts in Social Gerontology: current literature on Ageing CINAHL EMBASE General Science Index PubMed Psychlit Wilson OmniFile Full Text Mega Edition link to index info here: https://academic.oup.com/gerontologist/pages/About)	PubMed
Ageing and society	(published by Cambridge core, – link here: https://www.cambridge.org/core/journals/ageing-and-society#)	Web of science
Science, technology and human values	published and indexed by SAGE link here: https://journals.sagepub.com/home/sth	Scopus, SAGE
Social studies of science	indexed by SAGE – link here: https://journals.sagepub.com/home/sss	Scopus SAGE
JMIR	JMIR is indexed in more than 18 bibliographic databases and abstracting services, including Medline [Index Medicus], PubMed, Directory of Open Access Journal (DOAJ Seal), CINAHL, Information Science Abstracts, INSPEC (Institution of Electrical Engineers), Communication Abstracts, The Informed Librarian Online, LISA (Library and Information Science Abstracts), EMBASE, Scopus, Science Citation Index Expanded, PsycINFO, CABI, LISTA (Library / Information Sciences & Technology Abstracts), ASSIA (Applied Social Sciences Index and Abstracts) database, CSA Social Services Abstracts database, and others. Link here: https://www.jmir.org/about-journal/indexing-and-impact-factor	PubMed, EMBASE, Scopus
Design studies	published by Elsevier meaning that it is indexed by science direct	Scopus
Technoscienza	index info here: http://www.tecnoscienza.net/index.php/tsj/search	Scopus
Science & technology studies	indexed by scopus and ebsco host	Scopus
Journal of aging studies	published by Elsevier and therefore indexed by science direct	Pubmed Scopus
Science as culture	published by taylor & francis, indexed by many link to full list here: https://www.tandfonline.com/action/journalInformation?journalCode=csac20).	Scopus

Societies	indexed by both scopus and web of science	Scopus/ web of science
Journal of responsible innovation	published by taylor & francis, indexed by scopus as well as web of science.	Scopus/ web of science
Archives of Gerontology and Geriatrics Research	Published by Elsevier, indexed by Scopus, PubMed/Medline, SCI, PsychINFO ++ (Science direct?)	Scopus, PubMed
Gerontology	International journal of experimental, clinical, behavioural, and technological gerontology. Published by Karger International. Indexed by PubMed, MEDLINE, web of science, PsychINFO, Scopus, Embase, CINAHL +++	PubMed Scopus
Journal of Technology in Human Services	Abstracted and/or indexed in: ASSIA (Applied Social Sciences Index & Abstracts); CSA; Dietrich's Index Philosophicus; EBSCOhost Online Research Databases; International Aerospace Abstracts; LISA: Library & Information Science Abstracts; Materials Business File; National Library of Medicine / PubMed; OCLC / ArticleFirst; Ovid / Inspec; PsycFIRST; PsycINFO / Sociological Abstracts; Scopus; Social Work Abstracts; Solid State and Superconductivity Abstracts; Studies on Women and Gender Abstracts; Thomson Reuters Emerging Sources Citation Index; and World Ceramics Abstracts (Online).	Scopus
Journal of aging and social policy		PubMed
Medical anthropology: cross-cultural studies in health and illness		Scopus
Disability and rehabilitation: Assistive technology		Scopus PubMed
